# SAMPL7 physical property prediction from EC-RISM theory

**DOI:** 10.1007/s10822-021-00410-9

**Published:** 2021-07-19

**Authors:** Nicolas Tielker, Stefan Güssregen, Stefan M. Kast

**Affiliations:** 1 Physikalische Chemie III, Technische Universität Dortmund, Otto-Hahn-Str. 4a, 44227 Dortmund, Germany; 2 Sanofi-Aventis Deutschland GmbH, R&D Integrated Drug Discovery, 65926 Frankfurt am Main, Germany

**Keywords:** SAMPL, Distribution coefficient, Solvation model, Quantum chemistry, Integral equation theory, EC-RISM

## Abstract

Inspired by the successful application of the embedded cluster reference interaction site model (EC-RISM), a combination of quantum–mechanical calculations with three-dimensional RISM theory to predict Gibbs energies of species in solution within the SAMPL6.1 (acidity constants, p*K*_a_) and SAMPL6.2 (octanol–water partition coefficients, log *P*) the methodology was applied to the recent SAMPL7 physical property challenge on aqueous p*K*_a_ and octanol–water log *P* values. Not part of the challenge but provided by the organizers, we also computed distribution coefficients log *D*_7.4_ from predicted p*K*_a_ and log *P* data. While macroscopic p*K*_a_ predictions compared very favorably with experimental data (root mean square error, RMSE 0.72 p*K* units), the performance of the log *P* model (RMSE 1.84) fell behind expectations from the SAMPL6.2 challenge, leading to reasonable log *D*_7.4_ predictions (RMSE 1.69) from combining the independent calculations. In the post-submission phase, conformations generated by different methodology yielded results that did not significantly improve the original predictions. While overall satisfactory compared to previous log *D* challenges, the predicted data suggest that further effort is needed for optimizing the robustness of the partition coefficient model within EC-RISM calculations and for shaping the agreement between experimental conditions and the corresponding model description.

**Supplementary Information:**

The online version contains supplementary material available at 10.1007/s10822-021-00410-9.

## Introduction

For more than a decade the SAMPL blind prediction challenges (Statistical Assessment of Modeling of Proteins and Ligands) [1] represent an optimal testbed for evaluating and optimizing the performance of computational models to predict experimental reference data. Our group participated in the past in a number of challenges on small molecule physicochemical properties, starting with SAMPL2 on tautomerization free energies in water [2], SAMPL5 on cyclohexane-water distribution coefficients (log *D*_7.4_) [3], SAMPL6.1 on aqueous p*K*_a_ values [4], and SAMPL6.2 on octanol–water partition coefficients (log *P*) [5]. The methodology employed throughout was the embedded cluster reference interaction site model (EC-RISM) developed by us on the basis of combining three-dimensional (3D) RISM theory [6–8] as a solvation model with quantum–mechanical (QM) calculations [9]. This computational model allows for the calculation of Gibbs energies of species in solution that can be combined in thermodynamic cycles to yield derived quantities such as the previous SAMPL challenge targets mentioned above. The challenges themselves triggered further development of the model in terms of identifying and optimizing methodical details throughout the history, as has been discussed in broad detail in a recent overview paper [10]. Briefly summarizing the key results, we expect a p*K*_a_ accuracy on the order of 1 and octanol–water log *P* accuracy below 1 p*K* units. log *D*_7.4_ values at the pH given as subscript have only been computed thus far for cyclohexane-water distributions, yielding expected errors on the order of 2 p*K* units, despite considerably better performance of the underlying p*K*_a_ and log *P* models. This finding even holds for a re-evaluation of the older SAMPL5 dataset with the most highly optimized EC-RISM setup, giving rise to speculations about fundamental inconsistencies of the computational representation of experimental reality [10]. These issues have not been resolved yet as related but methodically different QM-based log *D* models typically exhibit similar error margins.

The latest SAMPL7 physical property challenge [11] represents a continuous further development, as participants were this time asked to predict both aqueous p*K*_a_ values similar to SAMPL6.1 and octanol–water partition coefficients, log *P*, as during SAMPL6.2. Both quantities could be combined in the usual way to compute log *D*_7.4_ values. Experimental reference data on these quantities have been provided after the submission deadline although these were not part of the challenge. Based on our earlier experiences we decided to essentially apply our established models from SAMPL6.1 and 6.2 [4, 5]. Slight variations to be described below were not projected to influence the expected performance. As will be demonstrated, the performance of the acidity model even surpassed expectations while the partition coefficient results were significantly worse than found before for both training and SAMPL6.2 test set data, merging to an overall still satisfactory result for log *D*_7.4_ predictions. This inspired us in the post-submission phase to generate a new set of conformations to be tested as a potential source of uncertainty. Results of the original submission and the variation including consensus values are discussed in the following, also in comparison with data from other participants who submitted both p*K*_a_ and log *P* predictions.

## Computational details

As the RISM solvation Gibbs energy parametrizations for water and octanol as well as the optimized p*K*_a_ model were taken from previous SAMPL challenges [4, 5] (with one minor adjustment for octanol described below), we here focus on comparing the different schemes for generating conformations of the challenge compounds that had been employed in the past.

For the submission, the workflow originally developed during the SAMPL5 challenge was applied to all microstates, including the additional relevant microstates complementing the set during the submission phase [1, 3, 10]. For each individual microstate, 200 conformations were generated starting from the original structures with the EmbedMultipleConfs utility of RDKit [12, 13]. If the molecule contained fewer than 7 rotatable bonds only 50 conformations were generated instead to reduce the computational cost for compounds with less conformational degrees of freedom. All conformations generated this way were optimized using the antechamber tool of the Amber12 suite [14], parametrized with AM1-BCC charges and GAFF version 1.7 parameters for bonded and non-bonded terms [14–17]. Solvation in water and octanol was simulated using an ALPB implicit solvation model with dielectric constants of 78.5 for water and 9.86294 for octanol, yielding two separate sets of 50 or 200 conformations each [18]. After the optimization an energy-filtered structural root mean square differences (RMSD) based clustering was applied to reduce the number of conformations to a more manageable number. Structures with a force field energy 20 kcal mol^−1^ above the apparent global minimum structure of a given microstate were discarded, with the minimum structure seeding the first cluster. All other conformations were then compared to the minimum structure in the order of increasing force field energies by using the GetBestRMS function of RDKit to calculate the RMSDs. If a structure had an RMSD of less than 0.5 Å it was discarded, while structures with a larger RMSD were added as additional cluster representatives. The resulting cluster representatives were optimized quantum-chemically using the B3LYP/6–311 + G(d,p)/IEFPCM level of theory implemented in Gaussian 16 Rev. C.01 [19]. After the quantum-chemical optimization another purely RMSD-based clustering using a cutoff of 0.5 Å was employed to remove conformations that reached the same minima during optimization. Up to five conformations with the lowest quantum-chemical energy were used in EC-RISM calculations to determine the Gibbs energy in solution per microstate by computing a partition function average. The compounds’ microstate Gibbs energies in the respective solvents $G_{t}^{{{\text{sol}}}}$ was computed with the approach used in the SAMPL6 log *P* challenge by taking the sum of the electronic energy of the polarized wave function $E_{tc}^{{{\text{sol}}}}$ and the corrected excess chemical potential $\mu_{{tc{\text{,corr}}}}^{{{\text{ex}}}}$ of all conformations *c* per microstate *t* as

1 $$G_{t}^{{{\text{sol}}}} = - \beta^{ - 1} \ln \sum\limits_{c} {\exp [ - \beta (E_{tc}^{{{\text{sol}}}} + \mu_{{tc{\text{,corr}}}}^{{{\text{ex}}}} )]}$$

with $\beta = (RT)^{ - 1}$ representing an inverse temperature. Detailed descriptions of how the electronic energies and excess chemical potentials are calculated and the specific corrections used for water and octanol can be found in previously publicized works [3–5]. The partition coefficient then follows from

2 $$\log P = - \frac{{\Delta_{{{\text{trans}}}} G^{0} }}{RT\ln 10} = \frac{{G_{{{\text{wat}}}}^{0} - G_{{{\text{oct}}}}^{0} }}{RT\ln 10}$$

with

3 $$G^{{0,\{ {\text{wat}}|{\text{oct}}\} }} = - \beta^{ - 1} \ln \sum\limits_{t} {\sum\limits_{c} {\exp [ - \beta (E_{tc}^{{{\text{sol,}}\{ {\text{wat}}|{\text{oct}}\} }} + \mu_{{tc{\text{,corr}}}}^{{{\text{ex,}}\{ {\text{wat}}|{\text{oct}}\} }} )]} }$$

After the original submission, the conformer generation approach used during the SAMPL6 challenges was also applied to the microstates of the SAMPL7 challenge to investigate if another set of conformations yields different results [4, 5]. In this case we generated the initial structures for QM optimization by using a force field-based sampling procedure. Structures of each microstate were taken as SMILES strings provided by the organizers. The flipper utility that is part of Omega [20] was used to perform a full enumeration of stereoisomers (i.e. generation of both formal E/Z isomers in cases they were not specified in the SMILES string), and initial 3D coordinates were generated using Corina [21]. For compounds bearing a sulfoxide moiety, additional stereoisomers with inverted chirality at the sulfur atom were added manually. The subsequent conformational analysis of all states was performed using Maestro 12.5 and Macromodel 12.9 as included in the 2020–3 release of the Schrödinger software suite [22]. Default parameters were used unless stated otherwise. We used the mixed torsional/low-mode conformational search algorithm and employed the OPLS3 force field in conjunction with an implicit water model. Conformational search up to a maximum of 1000 steps was carried out with 100 steps per rotatable bond present in the microstate. For saving conformations an energy window of 5 kcal mol^−1^ was used and redundant conformations were eliminated based on a RMSD cutoff of 1.5 Å. All resulting microstate conformations were forwarded to QM-based geometry optimization on the B3LYP/6–311 + G(d,p)/IEFPCM level of theory, and again up to 5 highest-ranking (lowest free energy) structures were selected for further processing by EC-RISM. Unlike the RDKit-based workflow employed for submission where different conformational sets for water and octanol were obtained und reoptimized, the sampling approach yielded only one set of conformations representative for water while final structural ensembles again differed slightly between solvents due to IEFPCM optimization mimicking the respective water and octanol environments.

For the EC-RISM calculations similar settings and solvent susceptibilities to those used in the SAMPL6 log *P* challenge were employed here to calculate the Gibbs energies of the compounds in solution, with one minor adjustment already pointed out as a perspective in our SAMPL6.2 paper [5]. Here, the water-saturated octanol solvent susceptibility was generated using the experimental number densities of 1.3598·10^–3^ Å^−3^ for water and 3.65787·10^–3^ Å^−3^ for octanol sites, and a dielectric permittivity of 8.41. As discussed in the original paper this is not expected to lead to significant deviations from the original water-saturated octanol model. Parametrization results and slightly changed resulting parameters for correcting the RISM excess chemical potential are shown in Fig. S1 and Table S1 in Online Resource (OR) 1. The 3D RISM calculations were conducted utilizing the PSE-2 closure [23] for water and the PSE-1 (Kovalenko-Hirata) closure for octanol. The RISM equations were solved on a cubic periodic grid of fixed size consisting of 128^3^ grid points and 0.3 Å grid spacing. The partial molar volumes entering the free energy correction expression [5] were calculated with the experimental compressibility of 0.761·10^–9^ Pa^−1^ for octanol and the 1D RISM estimate of the isothermal compressibility of 0.717062·10^–9^ Pa^−1^ for water [18, 24] from the total correlation function route. All EC-RISM calculations were done using the MP2/6–311 + G(d,p) level of theory within Gaussian 09 Rev. E.01 [25] using exact electrostatics taken directly from the wave function [4]. As in previous works, a more recent version of Gaussian was used for optimizations to take advantage of performance improvements [3, 5].

Aqueous p*K*_a_ values were calculated from the optimized model developed in our SAMPL6.1 publication [4] for each pair of microstates separated by one unit charge difference and transformed, along with tautomer Gibbs energy differences, to the standard reaction free energy format required by the organizers by referring to a specific microstate reference [11]. As will be shown elsewhere in the SAMPL7 overview paper [26], the transformation from microstate p*K*_a_ values (or corresponding standard reaction free energies) to the macrostate p*K*_a_ values is equivalent to the “state transition” (ST) formalism analyzed by us recently [27, 28], so these values were submitted along with the microstate standard reaction free energies from microstate-specific Gibbs energies calculated according to Eq. (1). In the following we also compare these results to the “partition function” (PF) approach [27] using the same input data for state Gibbs energies. Gas phase energies were not needed, neither for p*K*_a_ nor for log *P* calculations, as these cancel exactly because the gas phase ensembles of compounds evaporating from the water or the octanol phases are identical [10]. Finally, log *D*_7.4_ predictions were derived from calculated p*K*_a_ and log *P* data in the usual way [3, 10].

## Results and discussion

### General outline and p*K*_a_ predictions

We not only present our own data but also try to put the results into context by comparison with other participants. Here we chose only those submissions for which the final quantity, log *D*_7.4_ could in principle be calculated, i.e. challenge contributions containing both, ranked p*K*_a_ and log *P* predictions. Without going into methodical detail, the following 5 submissions satisfied the conditions, termed according to the submission nomenclature (1) “MD (CGenFF/TIP3P)|Gaussian_corrected”, (2) “TFE-SMD-solvent-opt|DFT_M06-2X_SMD_explicit_water”, (3) “TFE-NHLBI-TZVP-QM|TZVP-QM”, (4) “TFE IEFPCM MST|IEFPCM/MST”, (5) “TFE b3lypd3|DFT_M05-2X_SMD” [11]. The first part in front of the pipe symbol refers to the log *P* model, the second to the p*K*_a_ approach. Accordingly, our own models are termed (0) “EC_RISM_wet|EC_RISM”. As outlined in the preceding section, besides data from the original structure set (“orig”) we also report results from the new set of geometries (“new”) separately and from a combination (“comb”) by simply augmenting the microstate partition function with the new energies, ignoring the possibility of duplicates. In the following analysis of acidity constants, the state transition approach [27] was used for deriving macroscopic p*K*_a_ values from submitted free energies throughout for all submissions.

**Fig. 1:**
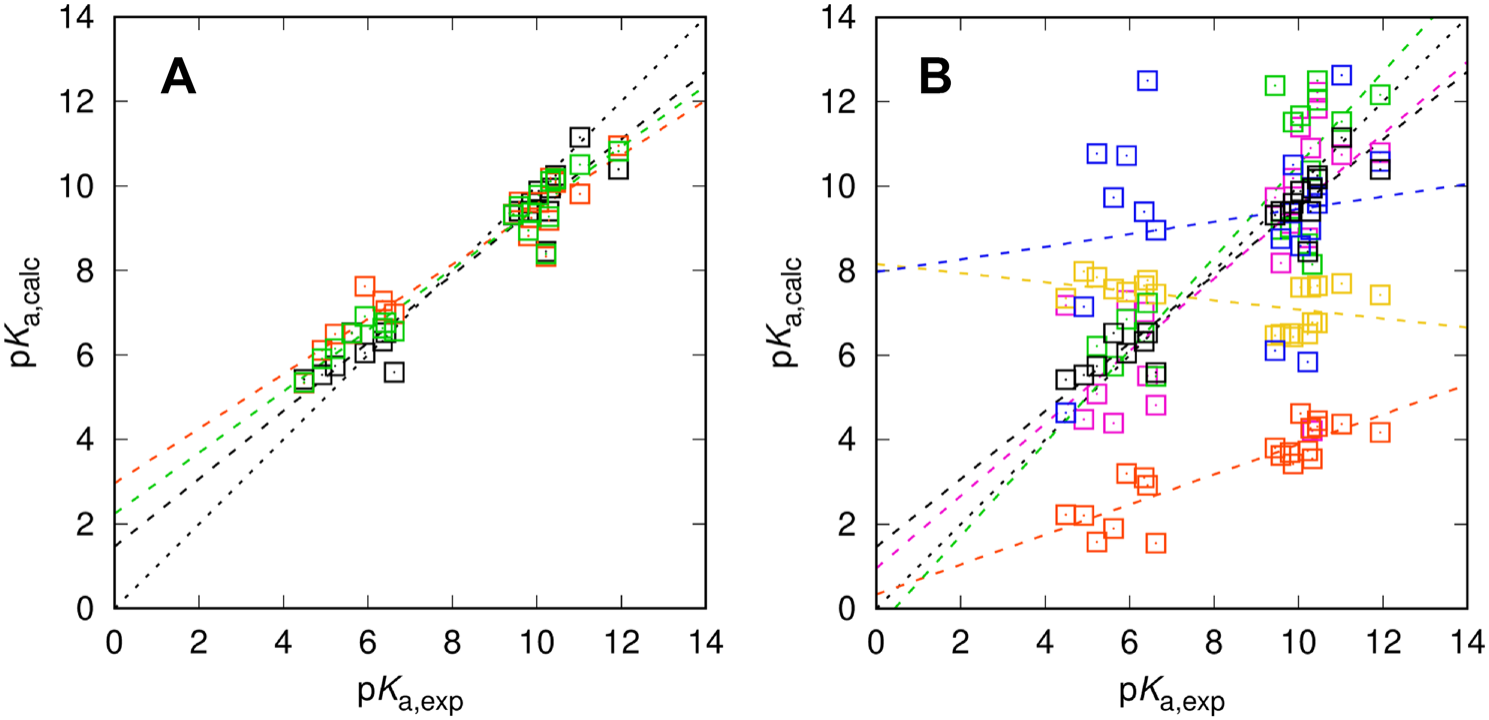
Macroscopic acidity constants for the SAMPL7 set calculated using EC-RISM (**A**) and other models discussed in this work (**B**) with different sets of conformations in panel (**A**) encoded by symbol and line colors, original: black, (0) EC_RISM (orig); new: orange, (0) EC_RISM (new); combined: green, (0) EC_RISM (comb). The model comparison in panel (B) is color-coded as black: (0) EC_RISM (orig), orange: (1) Gaussian_corrected, green: (2) DFT_M06-2X_SMD_explicit_water, yellow: (3) TZVP-QM, magenta: (4) IEFPCM/MST, blue: (5) DFT_M05-2X_SMD. Two data points of submission (2) DFT_M06-2X_SMD_explicit_water are not shown in panel (**B**) as they lie far outside the experimental range. Dashed lines indicate descriptive linear regression results. Raw data are provided as OR3 for structures and OR4 for energies. Macroscopic p*K*_a_ values for other participants’ models were taken from the SAMPL7 repository [11] and are additionally collected in OR7

All p*K*_a_ models agreed in the choice of the relevant ionization state change related to the observed macroscopic p*K*_a_ values, going from a neutral acid to a negatively charged base, which greatly simplified the analysis. Transitions from charged acids were accompanied throughout by negative p*K*_a_ predictions and could be ignored for comparison with experiment. Results for macroscopic acidity constants are shown in Table 1 and Fig. 1 with individual compound data summarized in Table 2. Apparently, EC-RISM outperformed other methods, exceeding expectations from earlier challenges and the training set performance (ca. 1 p*K* unit RMSE) with a submission RMSE of 0.72 p*K* units. High correlation measured by *R*^2^ and a regression slope near one, small absolute and signed errors indicate an overall robust model. The new set of conformations performed slightly inferior, though still in line with the metrics of the original set and not overlapping with prediction statistics of other models. Somewhat unexpectedly it turned out that the combined set of conformations did not lead to improvement. This means that the new conformations do not fully overlap with the old ones but add some new low-energy structures to the partition function that yield larger deviations in terms of their p*K*_a_ performance. The only conclusion at this point is that the observed discrepancy between different conformation sets can be taken as a measure of model uncertainty (not to be confused with expected prediction uncertainty).

**Table 1:** Statistical metrics for predicted acidity constants p*K*_a_ (root mean square error RMSE, mean absolute error MAE, mean signed error MSE, slope *m′*, intercept *b′*, and coefficient of determination *R*^2^ from descriptive regression) using EC-RISM and the other models discussed in this work

Model	RMSE	MAE	MSE	*m*′	*b*′	*R*^2^
(0) EC_RISM (orig)	0.72	0.53	− 0.20	0.80	1.46	0.93
(0) EC_RISM (new)	0.94	0.80	− 0.02	0.65	2.96	0.92
(0) EC_RISM (comb)	0.76	0.62	− 0.09	0.72	2.24	0.95
(1) Gaussian_corrected	5.36	5.12	− 5.12	0.35	0.33	0.76
(2) DFT_M06-2X_SMD_explicit_water	5.12	2.56	0.35	1.10	− 0.47	0.20
(3) TZVP-QM	2.90	2.75	− 1.20	− 0.11	8.16	0.23
(4) IEFPCM/MST	1.82	1.30	0.25	0.86	0.96	0.56
(5) DFT_M05-2X_SMD	2.90	2.28	0.78	0.15	7.97	0.03

**Table 2:** Experimental and calculated data for individual compound p*K*_a_ values from the different EC-RISM-based approaches

Compound	p*K*_a,exp_	p*K*_a,calc_(orig, PF)	p*K*_a,calc_(orig, ST)	p*K*_a,calc_(new, ST)	p*K*_a,calc_(comb, ST)
SM25	4.49	5.42	5.42	5.33	5.36
SM26	4.91	5.53	5.53	6.11	5.91
SM27	10.45	10.17	10.17	10.13	10.16
SM28^a^	–	13.95	13.95	14.38	14.30
SM29	10.05	9.88	9.88	9.61	9.78
SM30	10.29	9.40	9.40	9.18	9.27
SM31	11.02	11.15	11.15	9.81	10.50
SM32	10.45	10.25	10.25	10.08	10.18
SM33^a^	–	9.80	9.80	9.62	9.63
SM34	11.93	10.40	10.40	10.95	10.82
SM35	9.87	9.59 (9.592)^b^	9.59 (9.588)^b^	9.22	9.36
SM36	9.80	9.41	9.41	8.85	8.97
SM37	10.33	9.94 (9.944)^b^	9.94 (9.941)^b^	10.19	10.11
SM38	9.44	9.31	9.31	9.33	9.32
SM39	10.22	8.45	8.45	8.33	8.39
SM40	9.58	9.40	9.40	9.62	9.51
SM41	5.22	5.74	5.74	6.49	6.15
SM42	6.62	5.59	5.59	6.97	6.56
SM43	5.62	6.52	6.52	6.49	6.52
SM44	6.34	6.32	6.32	7.28	6.63
SM45	5.93	6.05	6.05	7.63	6.91
SM46	6.42	6.52	6.52	7.05	6.76

^a^No experimental data available

^b^Numbers in parenthesis indicate results from more than 2 decimal figures in raw Gibbs energy data whereas all other numbers resulted from the original submission format restriction

Individual compound data in Table 2 further illustrates the prediction balance, with the largest deviation between prediction and experiment on the order of 1.6 p*K* units found for SM34 and SM39. For completeness, we there also show results from applying the partition function approach [27] which – as expected – only marginally differs from the state transition results.

### log *P* and log *D*_7.4_ predictions

**Fig. 2:**
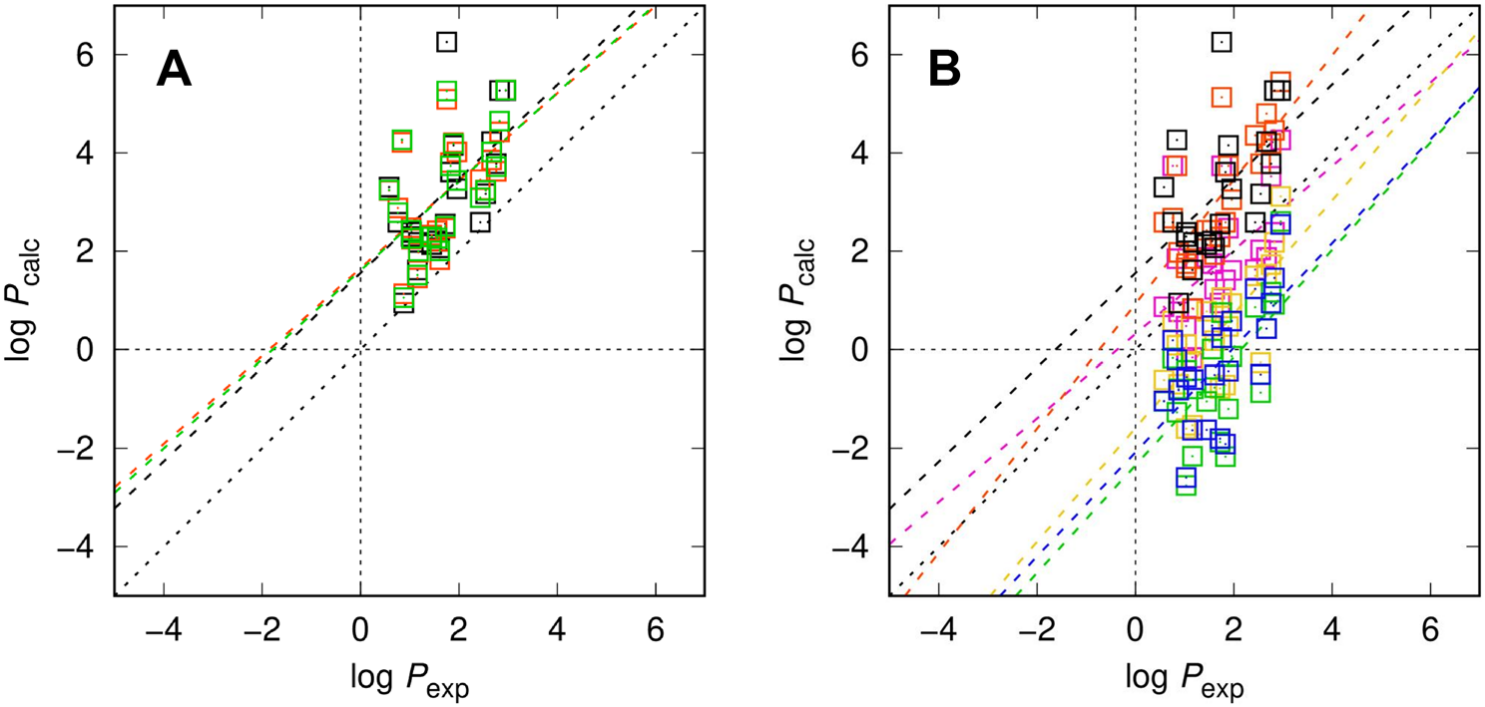
Partition coefficients for the SAMPL7 set calculated using EC-RISM (**A**) and other models discussed in this work (**B**) with different sets of conformations in panel (**A**) encoded by symbol and line colors, original: black, (0) EC_RISM_wet (orig); new: orange, (0) EC_RISM_wet (new); combined: green, (0) EC_RISM_wet (comb). The model comparison in panel (**B**) is color-coded as black: (0) EC_RISM_wet (orig), orange: (1) MD (CGenFF/TIP3P), green: (2) TFE-SMD-solvent-opt, yellow: (3) TFE-NHLBI-TZVP-QM, magenta: (4) TFE IEFPCM MST, blue: (5) TFE b3lypd3. Dashed lines indicate descriptive linear regression results. The signs of log *P* predictions for model (3) TFE-NHLBI-TZVP-QM have been inverted as accidentally the wrong reaction direction has been submitted. The log *P* values for other participants’ models were taken from the SAMPL7 repository [11]. Raw data are provided as OR5 for structures and OR6 for energies and are additionally collected in OR7

**Fig. 3:**
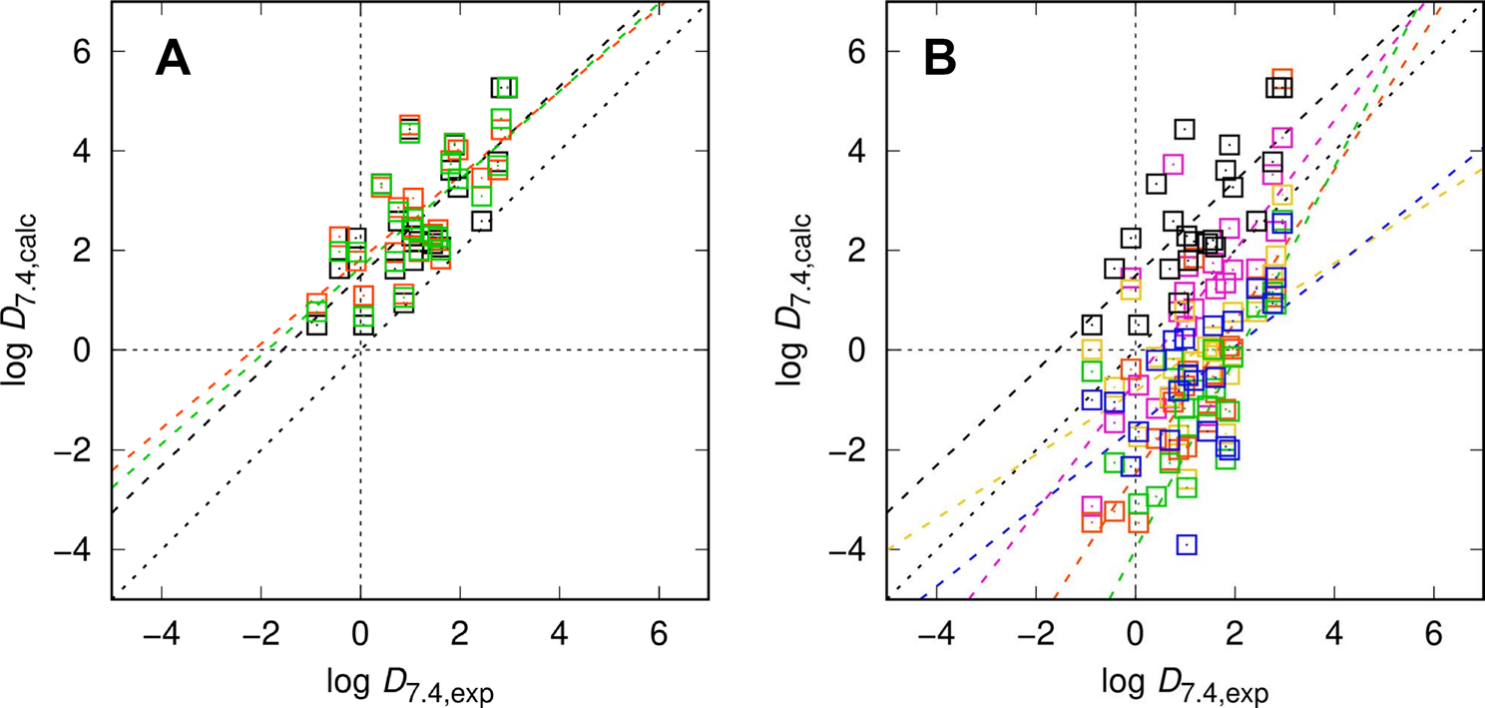
Distribution coefficients for the SAMPL7 set calculated using EC-RISM (**A**) and other models discussed in this work (B) with different sets of conformations in panel (**A**) encoded by symbol and line colors, original: black, (0) EC_RISM_wet (orig); new: orange, (0) EC_RISM_wet (new); combined: green, (0) EC_RISM_wet (comb). The model comparison in panel (B) is color-coded as black: (0) EC_RISM_wet (orig), orange: (1) MD (CGenFF/TIP3P)|Gaussian_corrected, green: (2) TFE-SMD-solvent-opt|DFT_M06-2X_SMD_explicit_water, yellow: (3) TFE-NHLBI-TZVP-QM|TZVP-QM, magenta: (4) TFE IEFPCM MST|IEFPCM/MST, blue: (5) TFE b3lypd3|DFT_M05-2X_SMD. Dashed lines indicate descriptive linear regression results. One data point of submission (2) TFE-SMD-solvent-opt|DFT_M06-2X_SMD_explicit_water is not shown in panel (**B**) as it lies far outside the experimental range. The signs of underlying log P predictions for model (3) TFE-NHLBI-TZVP-QM|TZVP-QM have been inverted as accidentally the wrong reaction direction has been submitted. Calculated data are provided as OR7

Given the successful application of the EC-RISM model to octanol–water phase partitioning of neutral compounds during SAMPL6.2 [5] (training and test set RMSEs of ca. 1.5 and 0.5 p*K* units), we expected similar performance for the SAMPL7 compound set. However, numbers reported in Tables 3 (statistical metrics) and 4 (individual compound data) and illustrated in Figs. 2 and 3 for log *P* and log *D*_7.4_, respectively, show a satisfactory, yet worse than expected overall result. With a log *P* RMSE for the original conformations of 1.84 p*K* units the upper limit of our expectation was slightly exceeded, and the non-zero MSE and regression intercept indicates a systematic trend to overestimate log *P* values, which has not been observed with our models before. Adding new conformations here somewhat improves the results, unlike the p*K*_a_ case, but not to an extent that we would assume to have pinpointed the origin of the discrepancies. It is possible that the specific chemistry of the SAMPL7 set is so different from earlier datasets tested that our model development is not yet robust enough to capture very diverse systems. One candidate for deeper investigation is the element sulfur which is not well represented in our reference datasets and which could have implications for the chosen theoretical level of theory, most likely the basis set.

**Table 3:** Statistical metrics for partition (log *P*) and distribution coefficient predictions (log *D*_7.4_) (root mean square error RMSE, mean absolute error MAE, mean signed error MSE, slope *m′*, intercept *b′*, and coefficient of determination *R*^2^ from descriptive regression) using EC-RISM and the other models discussed in this work

Model	RMSE	MAE	MSE	*m*′	*b′*	*R*^2^
*log P*						
(0) EC_RISM_wet (orig)	1.84	1.49	1.49	0.96	1.56	0.29
(0) EC_RISM_wet (new)	1.73	1.47	1.47	0.89	1.65	0.33
(0) EC_RISM_wet (comb)	1.72	1.45	1.45	0.90	1.61	0.33
(1) MD (CGenFF/TIP3P)	1.63	1.41	1.38	1.26	0.93	0.54
(2) TFE-SMD-solvent-opt	2.39	2.19	− 2.19	1.09	− 2.35	0.40
(3) TFE-NHLBI-TZVP-QM	1.55	1.34	− 1.34	1.16	− 1.59	0.52
(4) TFE IEFPCM MST	1.03	0.80	0.07	0.85	0.32	0.27
(5) TFE b3lypd3	2.19	1.98	− 1.98	1.06	-2.08	0.40
*log D*_*7.4*_						
(0) EC_RISM (orig)	1.69	1.43	1.43	0.95	1.49	0.53
(0) EC_RISM (new)	1.82	1.62	1.62	0.85	1.81	0.53
(0) EC_RISM (comb)	1.73	1.52	1.52	0.88	1.66	0.55
(1) Gaussian_corrected	2.27	2.13	− 1.84	1.53	− 2.49	0.62
(2) DFT_M06-2X_SMD_explicit_water	4.54	2.92	-2.88	1.92	-4.00	0.25
(3) TZVP-QM	1.72	1.47	− 1.26	0.64	− 0.82	0.25
(4) IEFPCM/MST	1.27	0.98	− 0.24	1.31	− 0.62	0.55
(5) DFT_M05-2X_SMD	2.15	1.78	− 1.78	0.80	− 1.54	0.32

For log *D* entries only the p*K*_a_ part of the model string is given. For compounds SM28 and SM33 where no experimental p*K*_a_ value was assigned and reported experimental log *P* and log *D*_7.4_ are identical, we assumed a hypothetically predicted log *D*_7.4_ to equal to predicted log *P*. The signs of log *P* predictions for model (3) TFE-NHLBI-TZVP-QM have been inverted as accidentally the wrong reaction direction has been submitted

Compared to the other log *P* models analyzed in this work our results rank average, with the best performing model (4) yielding an RMSE of ca. 1 p*K* unit. However, all models analyzed, including our own, show very little degree of correlation measured by *R*^2^, despite relatively reasonable regression slopes. This can be clearly traced back to a number of substantial outliers (e.g. SM42, SM43, see Table 4), for which there is no apparent explanation. The RMSE-wise best model (4) yields even a smaller value for this metric than ours, hinting at the possibility that chance plays a large role for obtaining good results.

**Table 4:** Experimental and calculated data for individual compound log *P* and log *D*_7.4_ values from the different EC-RISM-based approaches. “log *D*_7.4,exp_ (indirect)” denotes numbers reconstructed from experimental log *P* and p*K*_a_ values

Cmpd	log *P*_exp_	log *P*_calc_ (orig)	log *P*_calc_ (new)	log *P*_calc_ (comb)	log *D*_7.4,exp_	log *D*_7.4,exp_ (indirect)	log *D*_7.4,calc_ (orig)	log *D*_7.4,calc_ (new)	log *D*_7.4,calc_ (comb)
SM25	2.67	4.23	3.86	4.02	− 0.09	− 0.24	2.25	1.79	1.97
SM26	1.04	2.39	2.25	2.27	− 0.87	− 1.45	0.51	0.94	0.77
SM27	1.56	2.21	2.42	2.27	1.56	1.56	2.21	2.42	2.27
SM28	1.18	2.18	1.98	2.00	1.18	1.18	2.18^a^	1.98^a^	2.00^a^
SM29	1.61	2.07	1.83	2.01	1.61	1.61	2.07	1.83	2.01
SM30	2.76	3.78	3.63	3.72	2.76	2.76	3.78	3.62	3.71
SM31	1.96	3.27	4.02	3.44	1.96	1.96	3.27	4.02	3.44
SM32	2.44	2.59	3.46	3.09	2.44	2.44	2.59	3.46	3.09
SM33	2.96	5.27	5.28	5.28	2.96	2.96	5.27^a^	5.28^a^	5.28^a^
SM34	2.83	5.27	4.43	4.65	2.83	2.83	5.27	4.43	4.65
SM35	0.88	0.95	1.14	1.06	0.87	0.88	0.95	1.13	1.06
SM36	0.76	2.59	2.88	2.79	0.76	0.76	2.59	2.86	2.78
SM37	1.45	2.14	2.33	2.29	1.45	1.45	2.14	2.33	2.29
SM38	1.03	2.30	2.48	2.43	1.03	1.03	2.29	2.47	2.42
SM39	1.89	4.16	4.21	4.19	1.89	1.89	4.12	4.16	4.15
SM40	1.83	3.61	3.81	3.74	1.82	1.83	3.61	3.81	3.74
SM41	0.58	3.31	3.24	3.25	− 0.42	− 1.60	1.64	2.28	1.98
SM42	1.76	6.26	5.09	5.26	0.99	0.91	4.44	4.52	4.36
SM43	0.85	4.27	4.22	4.27	0.42	− 0.94	3.34	3.27	3.33
SM44	1.16	1.62	1.46	1.52	0.06	0.06	0.51	1.09	0.68
SM45	2.55	3.17	3.25	3.24	1.06	1.07	1.80	3.05	2.63
SM46	1.72	2.56	2.47	2.51	0.69	0.70	1.63	1.96	1.78

^a^For compounds SM28 and SM33 where no experimental p*K*_a_ value was assigned and reported experimental log *P* and log *D*_7.4_ are identical, we assumed a hypothetically predicted log *D*_7.4_ to equal to predicted log *P*. Applying our p*K*_a_ predictions for these compounds in order to convert log *P* to log *D*_7.4_ leaves the two decimals provided here unchanged

Results from log *D*_7.4_ predictions are slightly better, being even below our expectation of more than 2 p*K* units deviation with an RMSE of 1.69 p*K* units, ranking second (by a very small margin to the third) in the field of challenge participants with the best model (4) reaching 1.27. Here, adding new conformations again slightly worsened results due to weaker performance already observed for p*K*_a_ values. Scatter is, however, still large, so it is not possible to draw some general performance conclusions for this small and chemically focused dataset. One trend is obvious: Physics-based models such as those analyzed and compared in this work, that perform reasonably well and balanced in different prediction domains, will also perform well in combined model problems such as log *D*_7.4_ predictions. Still, log *D*_7.4_ remains a challenging property to be examined further in order to understand and improve model weaknesses. There is also room for improvement on the experimental side. We noted in some cases (see Table 4) that originally measured and reconstructed log *D*_7.4_ from p*K*_a_ and log *P* differ. Although there is apparently no correlation with prediction performance or failure, this could at least stimulate questions to further converge computational representations to match experimental reality.

## Concluding discussion

The most remarkable finding in this work is that apparently different conformational search or sampling strategies even for rather small molecules like those of the SAMPL7 set yield quite different results. Time did not permit deeper analysis of individual conformations, but it is clear that extended effort is needed for developing more consistent conformational sampling workflows. It is very likely that the problem originates already from the initial force field sampling stage as further QM-based optimization including a solvation model did not yield converged conformational ensembles.

However, our results show that conformational uncertainties alone are not responsible for the observed errors in thermodynamic quantities, which in our case imply an overestimated hydrophobicity. For water, results appear to be more reliable than for octanol, despite our earlier findings during SAMPL6.1 and SAMPL6.2 from which we expected better performance for log *P* than for p*K*_a_ predictions. In light of the different chemistry of SAMPL7 compared to SAMPL6 compounds, this hints at a possibly problematic description of sulfur-octanol interactions which could be related to the QM level of theory and/or sulfur-octanol dispersion interactions that are not modeled by first principle methods but by empirical Lennard–Jones terms. In the SAMPL7 challenge each compound contains a sulfone moiety whereas this functional group is represented by only one single MNSOL database entry, (sulfolane, *test2027*). This compound was predicted with an error of 4.83 kcal mol^−1^ for octanol, the largest in the entire training set [5]. For water the error is only 3.63 kcal mol^−1^, so it is likely that the error cancellation within the same solvent, as seen for the acid/base pair within p*K*_a_ predictions, does no longer apply for transfer free energies between different solvents. However, more solvent-specific experimental data, such as solvation free energies are necessary to confirm this hypothesis.

Another remarkable observation is that log *D* values taken directly from experiment or from a reconstruction based on experimental acidity and partition coefficients do not yield identical numbers in all cases. In cases where the two approaches differ significantly, i.e. for SM25, 26, 41–43, the reconstructed distribution coefficient is smaller, i.e. more negative than the direct measurement. This means that possibly a higher amount of the compound is dissolved in the aqueous phase than expected from neutral state partitioning alone if we take the reconstructed data as correct. If we, however, accept the direct experimental result then the opposite conclusion would emerge, namely that a larger compound fraction is dissolved in the organic phase. In other words, this could be interpreted as a missing contribution of charged species in the organic phase in our calculations where, via the standard formula for converting log *P* to log *D*, the presence of charged microstates in the nonaqueous phase is by definition excluded. This statement should in any case be viewed with caution as a range of alternative explanations could come into play, such as aggregation, nonideality effects due to insufficient dilution and so forth. However, observed inconsistencies are again a source and stimulus of deeper analysis including the correct agreement between experimental reality and its computational model representation.

## Supplementary Information

Below is the link to the electronic supplementary material.

Supplementary file1 (ZIP 1809 KB)
