# Supplementary material for: SAMPL7 physical property prediction from EC-RISM theory
Source: J Comput Aided Mol Des. 2021 Jul 19;35(8):933–41. doi: 10.1007/s10822-021-00410-9 (PMC8367877; doi:10.1007/s10822-021-00410-9)
Supplement: Supplementary file 1 — Supplementary file1 (ZIP 1809 KB) [file 10822_2021_410_MOESM1_ESM.zip › OR/OR1.pdf]

Online Resource 1

SAMPL7 physical property prediction from EC-RISM theory

Nicolas Tielker,† **Stefan Güssregen,***,‡ **Stefan M. Kast***,†

†Physikalische Chemie III, Technische Universität Dortmund, Otto-Hahn-Str. 4a, 44227 Dortmund, Germany

‡Sanofi-Aventis Deutschland GmbH, R&D Integrated Drug Discovery, 65926 Frankfurt am Main, Germany

*Correspondence should be addressed to S.M.K.
E-mail: stefan.kast@tu-dortmund.de, phone: +49 (231) 755-3906, ORCID ID: 0000-0001-7346-7064.

Table S1 Regression parameters of uncorrected and optimized ("2-par" using the terminology in Ref. [1]) EC-RISM/PSE-1-based Gibbs energy of water-saturated octanol-water solvation models (c_V / kcal mol^{-1} Å^{-3}) along with statistical metrics (root-mean-square error RMSE / kcal mol^{-1}, mean absolute error MAE / kcal mol^{-1}, mean signed error MSE / kcal mol^{-1}, slope m', intercept b' / kcal mol^{-1}, and coefficient of determination R^2 from descriptive regression). As in the SAMPL6.2 challenge [1], c_V corresponds to partial molar volumes computed using the total correlation function route with the experimental isothermal compressibility of $0.761\cdot10^9$ Pa^{-1} for octanol [2]. Raw data from current ("new") calculations are provided as Online Resource 2 (OR2), while the structures used and original SAMPL6.2 data have already been published in previous work for which resulting statistical metrics ("old") are only shown for comparison [1].

Solvent model	RMSE	MAE	MSE	m'	b'	R^2	c_μ	c_V
Uncorrected (old)	5.40	4.89	4.89	0.55	2.00	0.71	-	-
Uncorrected (new)	5.58	5.06	5.06	0.54	2.09	0.70	-	-
2-par (old)	1.51	1.16	-0.10	0.87	-0.93	0.86	1.2892	-0.01315
2-par (new)	1.51	1.16	-0.09	0.87	-0.92	0.86	1.2915	-0.01414

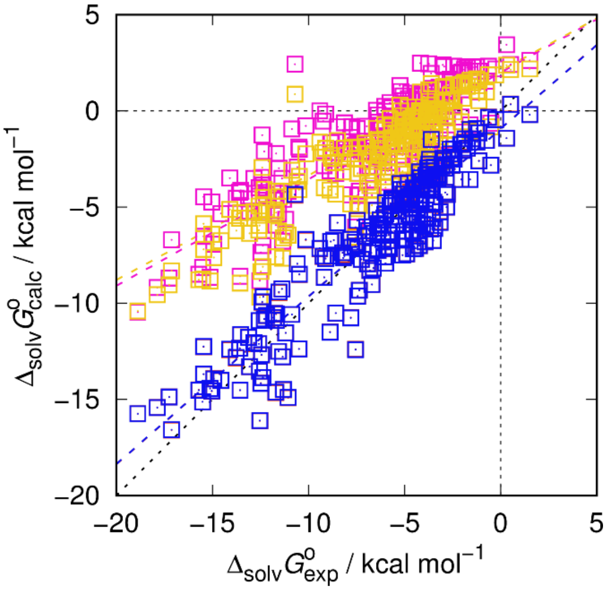

Fig. S1 Gibbs energies of solvation in water-saturated octanol from uncorrected and optimized EC-RISM calculations vs. the experimental results taken from the MNSOL database [3,4,5,6]. Uncorrected data is shown in yellow and magenta for the old and new solvent susceptibility, respectively, and corrected data in red (mostly overlaid by blue symbols) and blue. Dashed lines refer to linear regression statistics for old and new models, respectively while the dotted line (diagonal) denotes ideal correlation between prediction and experimental values.

References

1. Tielker N, Tomazic D, Eberlein L, Güssregen S, Kast SM (2020) J Comput-Aid Molec Des 34:453-461.
2. Matsuo S, Makita T (1989) Int J Thermophys 10:885-898.
3. Marenich AV, Kelly CP, Thompson JD, Hawkins GD, Chambers CC, Giesen DK, Winget P, Cramer CJ, Truhlar DG (2012) Minnesoate Solvation Database – version 2012, University of Minnesota, Minneapolis.
4. Kelly CP, Cramer CJ, Truhlar DG (2005) J Chem Theory Comput 1:1133-1152.
5. Marenich AV, Olson RM, Kelly CP, Cramer CJ, Truhlar DG (2007) J Chem Theory Comput 3:2011-2033.
6. Marenich AV, Cramer CJ, Truhlar DG (2009) J Phys Chem B 113:6378-6396.
